# The Treatment of Acute Gonococcic Urethritis in the Male1Read by invitation before the Scranton (Pa.) Clinical and Pathological Society

**Published:** 1909-05

**Authors:** James Pedersen

**Affiliations:** New York; Adjunct Professor of Genitourinary Surgery, New York Post-graduate Medical School and Hospital


### The Treatment of Acute Gonococcic Urethritis in the Male[1]

By JAMES PEDERSEN, M. D., New York.
Adjunct Professor of Genitourinary Surgery, New York Post-graduate Medical School and Hospital.

(*New York Medical Journal*, January 23, 1909.)

UNDER the steady pressure of the pen in the hands of those who, having given the subject much study and thought, have pleaded earnestly for a serious comprehension of a serious disease, the profession at large is at last yielding to the conviction that the long lived, subtle gonococcus is by far a more potent depopulator than the insidious cause of syphilis. That gonococcic urethritis is the greater menace to the body politic is becoming an universally admitted fact. The popular impression of the relative character of the two diseases has been revolutionised. No better summary of the facts need be given than that deduced by the late R. W. Taylor. Speaking through the pages of his still living book he says: "When we consider the vast range of pathological conditions which gonorrhea may cause or lead to, we are certainly warranted in asserting that it is, taken as a whole, one of the most formidable and far reaching infections by which the ruman race is attacked."

When the cause of a disease can be attacked and the method of its conveyance can be governed, the treatment of that disease has acquired not only a certain definiteness, but also a great importance, it having risen to the dignity of a preventive measure as well. The prevention of disease is the sublimest function of medicine. The cause of gonococcic urethritis is known and can be attacked; the method of its conveyance can be governed—at least to a great extent. Therefore, as physicians ever laboring for the physical, mental, and moral welfare of the race, it behooves us to arrest the progress and ravages of gonococcic urethritis, knowing well that thorough treatment of each individual case works for the prevention of this all too prevalent disease. This is our duty for the sake of the family and the state.

Let me open the subject proper of my lecture with the oft heard question: "What is the best treatment for gonorrhea?"—meaning gonococcic urethritis. I would reply by saying that no one of the advocated methods implied in the question can be named alone—much less heralded alone as the "best" treatment. I would make a point of this and emphasize it by adding, that no one method or set of means is applicable to every case of gonococcic urethritis. The "best" treatment, therefore, may be defined as that which consists of a rational application of all the

1. Read by invitation before the Scranton (Pa.) Clinical and Pathological Society advocated or admissible methods and means for combating this very serious disease, and their intelligent adaptation to the patient in hand. The "best" treatment is a *composite* treatment. If I may be allowed to quote from one of my previous papers, I shall briefly describe the several methods and their means, which may be said to form the component parts of the composite treatment. That done, I shall endeavor to apply them to the indications based on the present day knowledge of the biology and pathological anatomy of the disease. The several methods implied in the question may be classified as: 1, The expectant method; 2, the modified expectant method; 3, the hand injection method; 4, the irrigation method.

1. The expectant method places the patient under the best possible conditions for allowing the disease to run its normal course of from five to six weeks. The means are: absolute rest in bed, milk or milk and vichy diet, from one to two quarts of plain or mildly alkaline water a day, mild laxatives, rigid regulation of alcoholic beverages, tobacco, and coffee. The conventionalities of life, the average patient's uncompromising protest against having his business interrupted, not to mention the existence of a more scientific method for treating the disease, make this method as such an impossibility and a deficient method today. Its means, however, are rational means, and comprise all the essentials of the *general* treatment applicable to every case, as opposed to the purely *local* treatment. It quiets the circulation throughout the body; it puts the urethra at rest as far as possible, consistent with its function as a urinary canal; it maintains the urine in a bland state and supplies it in quantities sufficient to produce a frequent flushing of the urethra by physiological means without the possibility of traumatism—unless a stricture of small calibre is present as a complication.

2. The modified expectant method adds the administration of drugs for one or more of three purposes: (1) To influence the volume and reaction of the urine; (2) to render the urine more or less antiseptic; (3) to charge the urine with a medicament in solution which shall act upon the inflamed muocus membrane. For the first purpose there are the demulcents, the alkalies, sodium benzoate, sodium salicylate, salol, and saccharine. For the second purpose there are the several urinary antiseptics, of which urotropin, cystogen, lysidin, uriseptin, and helmitol serve as examples. For the third purpose, the balsams, of which copaiba, sandalwood, and cubebs remain the essential ones. Plain water in quantity is the best diluent. As to whether the reaction of the urine should be made alkaline or kept acid, there is a difference of opinion. Some authorities maintain that the alkalinity of the urine distinctly inhibits the growth of the gonococcus in the urethra, while others contend that the acidity of the urine prevents the gonococcus from invading the wall of the bladder. If the former view is accepted and alkalies are given, the tendency to ardor urinæ will be lessened, if not prevented, as an additional effect; if the latter view is accepted and the newer acidifying drugs are given (called the modern urinary antiseptics, of which urotropin is the type), the presence in the urine of the potent antiseptic, formalin, will be obtained as an additional advantage. As there exist, however, far more direct and conclusive means for not only inhibiting the gonococcus in its first culture chamber—the anterior urethra—but also for destroying it there, the feebly effective alkalies on the one hand, and the scarcely less feebly effective acidifying and formalin liberating drugs on the other, are not necessarily demanded. As the gonococcus invades by continuity of tissue through the intracellular spaces and lymphatics, medicaments that do not penetrate below the surface can have but a negative value. Any prevention of invasion of the bladder, attributed to the presence in it of acid urine containing formalin, has been more apparent than real; the gonococcus, even under favoring conditions, very rarely invades the bladder mucosa beyond the trigone. The drugs under immediate consideration may, therefore, be withheld until ardor urinæ develops and furnishes a positive indication for the alkalies, with or without a demulcent or a balsam. Even this indication can usually be met by the milk and vichy diet. Thus the patient's stomach may be spared considerable medication.

The balsam (sandalwood, copaiba, and cubebs) certainly render the urine less irritating, and, in spite of their stimulating effect upon the urethral mucous membrane (as indeed upon all mucous membranes) they contribute materially to the patient's comfort when ardor urinæ exists, even during the acutest stages of an acute urethritis, in all stages of which they are contraindicated theoretically. This happy effect is especially noticeable in acute posterior urethritis, from the mild up to the fairly severe grade; they promptly lessen the frequency and diminish the tenesmus to the great relief of the sufferer. But, because the balsams usually check the discharge (the symptom) without attacking the gonococcus (the cause), thus handicapping Nature's effort to rid the urethral tissues of the offending microörganism and misleading the patient into thinking he is already cured of what he is only too willing to regard as a trivial disease; because they tend to disorder the digestion, and, in full dose, to produce renal hyperemia, they should be withheld until needed as *adjuvants* in ardor urinæ or as *principals* in acute posterior urethritis.

3. The hand injection method brings prominently forward the present day medicinal means for directly attacking the gonococcus at the site of its invasion and development. Those means are the various silver albuminoid compounds, such as argonin, protargol, albargin, argyrol, and novargon. They represent the nearest approach yet made to specifics in gonococcic inflammation of the mucous membranes. That they exhibit so destructive an activity toward the gonococcus as to deserve the credit of having an affinity for it, and that they accomplish this destruction without, as yet, any appreciable damage to the mucous membrane, has been proved clinically to the satisfaction of many observers. They seem to fulfill Neisser's specifications for an ideal antibacterial agent for use in the urethra. As quoted by Finger, those specifications are: (1) it must kill the gonococcus; (2) it must not injure the mucous membrane; and (3) it must not increase the inflammation.

My personal experience with the silver albuminoid compounds has been confined to argonin, protargol, and argyrol. Following their use I have noted the disappearance of the gonococci and the subsidence of the discharge within from twenty-four hours to fourteen days, with few exceptions. When such an exception occurs, one or more conditions may be suspected and should be searched for as soon as possible or permissible: (1) a preëxisting focus of latent gonococci, such as a chronic vesiculitis, prostatitis, or Cowperitis affords; (2) oxaluria, or urine loaded with uric acid; (3) glycosuria; (4) tuberculosis as such or the diathesis.

The disappearance of the gonococci and the subsidence of the discharge within the first forty-eight hours—not an infrequent occurrence especially when the patient is seen in the very incipiency of the urethritis—is practically an abortion of the disease. A paragraph on this interesting and much desired effect is, therefore, appropriate at this point.

When silver nitrate was the only available means for this purpose, the fact was well recognised that any attempt to abort the disease was futile unless made at the very earliest stage. The discharge consists then only of a little serum and mucus, floating epithelium, and a very few pus cells, together with a few gonococci lying free or upon the epithelium. Relatively few patients were seen so early in the disease, therefore cases of true abortion were rare. Much more frequently the results amounted to an inhibition of the disease, and the end results were not always of the best, inasmuch as the cauterising effect of the silver nitrate solution (from 1 to 5 per cent.) used in the attempts, often left the patient with a deeply damaged mucous membrane, eventuating in stricture. These experiences led conservative genito-urinary surgeons to abandon attempts to abort the disease. Now, however, with the present potent but practically noncaustic silver albuminoid compounds, there exists the certainty of better results without the danger of subsequent stricture. In my experience with these compounds in solutions of suitable strength, the injection of two drachms into the interior urethra and their retention there for from five to ten minutes in the incipient stage. or stage of invasion, of a gonococcic infection of the urethra, will abort the acute stage of the disease and materially shorten its subsequent course, unless there is present a complicating sequel of an antecendent urethritis.

Convinced of the affinity of these compounds for the gonococcus, and admitting that they seem to possess the power of penetrating the mucous membrane and of attacking the gonococcus there, as well as on the surface, without damage to the mucous membrane, the direct, immediate, and prompt application of some one of them and its retention in contact with the urethral mucous membrane for from five to ten minutes as soon as gonococci have been demonstrated, appeals to me as a most rational method of treatment. It stands to reason that the more promptly the gonococcus is destroyed the fewer will be the complications and the "pathological conditions which it may cause." As a consequence, the shorter will be the course of the disease. The argument holds good, even though no more than an inhibition of the gonococcus is admitted as the maximum effect of this treatment. Not only is the method rational, but also very practical, when carried out by means of a hand syringe which the patient can use himself as frequently as directed. This entails a minimum of discomfort and loss of time. After having been taught how to apply the blunt tip of the hand syringe against the meatus to avoid leakage, and how to inject slowly and gently, the only accident he has to guard against is a staining of his clothing when releasing the solution from the urethra.

Another means of using these solutions is the familiar irrigator. To use it properly is an art that few patients can acquire within the time limit of its greatest utility. It is not possible for every patient to learn how to irrigate his anterior urethra without subjecting it to the traumatism of overdistention, nor how to avoid an inadvertent intravesical irrigation. The former causes pain; the latter exposes him to urethrocystitis and epididymitis. Taking another point of view, it is not easy to understand how the average patient can be taught to manage a quart of solution as conveniently, as expeditiously, and as effectively as he can a quarter of an ounce of a stronger and therefore more efficacious solution.

4. The irrigation method, that is, the copious flushing from the meatus of the anterior urethra alone or of the whole urethra with a warm solution of some medicament, has superseded and is an improvement upon the older retrograde irrigation by means of the various soft rubber and metal catheters and bulbous tipped irrigating instruments introduced into the urethra. There are today only two indications that justify instrumentation of an acutely inflamed urethra: (1) retention of urine, not yielding to all the lesser means for its relief, (2) extremely severe posterior urethritis—to which a definite reference will be made later. The means of applying the irrigations are a six ounce hand syringe with shield and adjustable tip, or a suitable glass nozzle and shield at the end of the tubing from an irrigator jar. By its advocates, the irrigation method is credited with results equal to those of the hand injection method. To my mind, however, the method is not as rational unless the medicament used be a silver albuminoid compound in weak solution, and unless the irrigation be given so gently and skilfully as not to subject the sensitive, acutely inflamed urethra to the additional traumatism of overdistention, nor force the fluid past the sphincter into the bladder. The intentional carrying out of intravesical irrigations during acute urethritis is irrational and unwarranted. They cannot but lead to hyperemia of the mucous membrane of the deep urethra. This in turn invites infection. The dangers, as already mentioned, are, urethrocystitis and epididymitis. The main utility of this method lies in the effect that moist heat has upon any inflammation. Hence, irrigations of the anterior urethra in conjunction with hand injections of one of the silver albuminoid compounds are to be employed in those neglected cases in which the inflammatory symptoms have become paramount, as shown by a red and edematous condition of the meatus, and a copious greenish yellow discharge. The irrigations should be discontinued as soon as these signs have disappeared.

It is a subject for regret and criticism that the irrigation method, or the irrigation treatment, as it is popularly known, should have been revived at the time when argonin, the first of the silver albuminoid compounds, was offered as a potent and supplanting substitute for the long list of sedative, antiseptic, and astringent injections, which for years had proved unsatisfactory and disappointing. It was this very disappointment, long continued and oft repeated, that had made the profession receptive of, in fact, eager for any change, the more radical the better, that bore a hint of promise in it. In spite of the fact that irrigation had been tried many years before and found wanting—so many years before that its negative results had been lost sight of—the "treatment" was resurrected and vaunted with the enthusiasm of originality. The sober judgment of the profession at large, taken unawares, was swept away. Attention was diverted from the truly potent and convenient argonin, and it was some time before its value was noticed by the general practitioner. Gradually, however, the results obtained with argonin became obvious. An item of great practical value from the view point of the patient is the fact that the hand injection method, as opposed to the irrigation method, does not oblige him to visit his physician from once to twice daily during the acute stage, nor to provide himself with specialised apparatus for use at home after preliminary instruction.

Such, in more or less detail, are the four applicable methods for the treatment of gonococcic urethritis. Before applying them to the several indications and molding them into what I have called a composite treatment, it will be in order to refer to the indications, based on the biology and pathology of the disease as understood today. What is gonococcic urethritis? It is a purulent inflammation of a delicate, highly sensitised mucous membrane, caused by a virulent, penetrating, long lived microorganism. The inflammation inflicts more or less damage upon one or more of the layers composing that mucous membrane and tends to chronicity. It follows, therefore, that the indications for treatment are: (1) the destruction of the gonococcus without increasing the damage done or being done to the mucous membrane; (2) the termination of the inflammatory process excited by and left behind by the gonococcus; (3) the repair of the damaged mucous membrane.

(1) *The destruction of the gonococcus without increasing the damage done or being done to the mucous membrane.* That the silver albuminoid compounds, in solutions not strong enough to cause any appreciable damage to the urethral mucous membrane, exert a potently destructive action on the gonococcus, cannot be denied. The clinical evidence is overwhelming. In a way that is unique they fulfill those requirements named by Neisser already quoted. They do kill the gonococcus, they do not injure the mucous membrane, they do not increase the inflammation. Therefore, they are employed as injections into the anterior urethra and are retained there for from five to ten minutes if the discharge contain gonococci. The same is done as a prophylactic measure if the history is at all suspicious, even though the microscopical examination, made on the instant, be negative. The patient is then taught how to use the injection for himself every three hours for the first twenty-four hours and every four hours thereafter. Every fourth day he reports for inspection, and each time a smear is examined. As the gonococci diminish in number, the strength of the injection is reduced, and the frequency of its use is gradually changed from four times daily to twice daily. After the gonococci have been absent for from three to seven days (depending upon the severity of the infection in the given patient), the injection is reduced to once a day. From five to ten days later (again depending upon the patient) it is discontinued altogether.

If the inflammatory signs of the disease are excessive at the outset or if they become so at any time during the course of this, the acute stage, the patient is provided with a tablet of bichloride of mercury one of which in eight ounces of water makes a solution of one in thirty thousand. He is to make a solution of that strength, using hot water, and with his one quarter ounce hand syringe he is to gently flush the anterior urethra six or eight times before each use of the silver albuminoid solution. If necessary, an additional and more copious irrigation is given daily or every other day by the physician.

(2) *The termination of the inflammatory process excited by and left behind by the gonococcus.* Almost any one of the mineral or vegetable astringents will meet this indication, provided *strong* solutions are avoided. The mineral astringents seem to have the preference. Zinc sulphate may be used up to gr. 2 in ℥i. Zinc sulphocarbonate up to gr. 5 in ℥i. Zinc iodide and zinc chloride, each, up to gr. ½ in ℥i. The astringent injection is used twice daily—rarely three times daily, at first. This frequency is gradually reduced as the catarrhal discharge diminishes, and when it has totally or practically disappeared, as shown by the presence of a mucoid morning drop at most, the injection in use is discontinued. If the catarrhal discharge persists longer than two weeks in a case of average severity, some complication—either antecedent or recent—is to be sought. Microscopical examinations of the catarrhal discharge are made regularly throughout this, the subacute stage, and if gonococci reappear, the silver albuminoid injection is at once resumed. The restrictions as to diet, alcohol, tobacco, coffee, and sexual excitement, both active and passive, are not abated; but a little more exercise is allowed.

(3) *The repair of the damaged mucous membrane.* Silver nitrate in solution is by far the best agent for this purpose, because the safest and most efficient. In solutions of from 1 to 5,000 to 1 in 250 it is brought into contact with the mucous membrane by means of the Ultzman syringe or (better) the Bangs's syringe sound. In solutions of from gr. 5 in ℥i up to gr. 10 in ℥i, it is applied by means of a cotton swab through the endoscope. Stronger than gr. 10 in ℥i (2 per cent.) is not advised. Every instrumentation should be carried out gently, and no form of instrumentation employed for this indication should be repeated oftener than once in five days. Once in seven days is often a safer average. The lubricant used should be soluble in water. Experience alone will teach when the treatment of this, the chronic stage, may be begun. Speaking generally, if the case has been one of mild infection and the previous history is negative as to stricture, the instrumental treatment may be delayed. If the contrary has obtained, especially if there is reason to suspect stricture—antecedent or recent—the treatment may be begun earlier—even before the morning drop has disappeared. Indeed, without judicious instrumentation—not repeated oftener than every five days—the catarrhal discharge will persist indefinitely in some cases. In such cases, as in all obstinate cases, prostatitis and seminal vesiculitis should be examined for.

When the morning drop persists in consequence of an unusual involvement of the urethral follicles (follicular urethritis) the irrigation method is of service as an adjuvant. The irrigations should be given to both the anterior and posterior portions of the urethra. This is now permissible, the chronic stage having been present for perhaps two to three weeks. The irrigating fluid, flowing from the meatus backward into the bladder, enters the follicles—their mouths being directed forward—and removes their retained secretions. The solution may be one to thirty thousand bichloride, full strength boric acid, 1 in 2,000 potassium permanganate of 1 per cent. (or less) of one of the silver albuminoid compounds.

For the sake of clearness, the treatment of *acute posterior urethritis* has not been mentioned thus far. It occurs in 90 per cent. of all cases of anterior urethritis and develops early in the course—on or about the eleventh day. The majority of cases give no symptoms. This fact should be kept in mind that the treatment of the posterior urethra is not overlooked or omitted while the treatment of the anterior urethra is being carried out during the chronic stage, for the repair of the damaged mucous membrane. A fair number of cases give, as symptoms of the acute invasion, more or less frequency alone, or frequency plus urgency, or frequency, urgency, and tenesmus.

As long as the tenesmus is not severe, what has been called the modified expectant treatment will be found sufficient. The patient should go to bed, his diet should be confined to milk and vichy, the alkalies or a balsam should be subscribed, and a saline laxative given if necessary. When the tenesmus has become unbearable and is uncontrollable by the other means, one of the two indications for instrumentation of an acutely inflamed urethra has arisen. The anterior urethra having been gently irrigated with warm boric solution, and anesthetised with a 2 per cent. solution of eucain, a soft rubber catheter, in size from 14 to 16 French, is passed and the deep urethra is gently flushed with two or three drachms of a silver albuminoid solution, or a solution of silver nitrate, in strength from 1 to 5,000 to 1 in 1,000. The relief is often surprisingly great and prompt. One such installation, or flushing, may prove sufficient to finally relieve the patient of his distressing symptoms. If, however, they recur—as they may after a day or two in cases of severe infection—the installation may be repeated. At the same time the possibility of a complicating acute prostatitis should be considered, and a prostatic abscess, as the cause of a frequent recurrence of the symptoms in spite of careful treatment, should not be overlooked.

20 East Forty-Sixth Street.

